# Utility and Pitfalls of β‐D‐Glucan for Diagnosis and Response Monitoring of Chronic Disseminated Candidiasis in Paediatric Cancer Patients

**DOI:** 10.1111/myc.70102

**Published:** 2025-08-09

**Authors:** Katharina F. Körholz, Marc T. Hennies, Heidrun Herbrüggen, Katja Krämer, Martina Ahlmann, Birgit Fröhlich, Frieder Schaumburg, Thomas Wiesel, Peter M. Rath, Andreas H. Groll

**Affiliations:** ^1^ Infectious Disease Research Program, Center for Bone Marrow Transplantation and Department of Pediatric Hematology and Oncology University Children's Hospital Münster Münster Germany; ^2^ Institute of Virology University Hospital Münster Münster Germany; ^3^ Institute of Medical Microbiology University Hospital Münster Münster Germany; ^4^ Vestische Kinder‐ Und Jugendklinik Datteln University of Witten‐Herdecke Witten Germany; ^5^ Institute of Medical Microbiology, University Hospital Essen, University of Duisburg‐Essen Essen Germany

**Keywords:** cancer, candidiasis, children, chronic disseminated candidiasis, diagnosis, monitoring, mycoses, treatment

## Abstract

**Background:**

β‐D‐Glucan (BDG) is a useful but nonspecific biomarker in patients with suspected invasive fungal diseases including *Pneumocystis* pneumonia. Little is known, however, about its utility for response monitoring in chronic disseminated candidiasis (CDC).

**Patients and Methods:**

We describe the utility and pitfalls of serum BDG in paediatric cancer patients with suspected CDC. BDG in serum was measured serially (i.e., 5 to 10 times of a time period of 200 to 400 days) by a commercially available assay (Fungitell; Associates of Cape Cod, MA, USA) and values were correlated to patient‐ and disease‐related variables.

**Results:**

Five paediatric patients (4f/1 m; 4–18 years) with acute lymphoblastic leukaemia (*n* = 4) and *Ewing* sarcoma (*n* = 1) followed between 2013 and 2024 were included. CDC was located in the spleen (*n* = 5), liver (*n* = 4), lungs (*n* = 3), CNS (*n* = 2), kidney (*n* = 1), and skin (*n* = 1); and diagnosed based on imaging, a positive blood culture (*n* = 1), a positive BDG assay in serum (*n* = 5), and absence of other etiologies. Patients received IV liposomal amphotericin B and/or caspofungin, followed by fluconazole orally for 184 to > 365 days, respectively. BDG concentrations in serum (35 time points) stayed elevated for prolonged periods of time, were independent of clinical symptoms, and returned to normal with resolution of imaging findings in the four leukaemia patients. In the patient with Ewing sarcoma, liver biopsy performed 5 months after diagnosis due to lack of improvement revealed disseminated aspergillosis.

**Conclusions:**

BDG in serum is useful for microbiological diagnosis and monitoring of probable CDC; however, it remains a non‐specific fungal biomarker whose results need to be scrutinised in patients who do not respond to treatment as expected.

## 1 Introduction

Invasive fungal diseases (IFDs) are important complications in immunocompromised paediatric cancer patients [1, 2]. Despite established algorithms for antifungal prophylaxis, the incidence rates can be as high as 12% in patients with hematologic malignancies and following allogeneic haematopoietic cell transplantation (HCT) [3, 4, 5, 6]. IFDs contribute to increased morbidity and mortality through their direct sequelae and delays in the control of the underlying malignancy [3]. In children with acute lymphoblastic leukaemia, one‐year mortality rates of up to 12.1% were observed, including 6.1% in yeast infections and 14.5% in mould infections [3]. Several factors have been identified that increase the risk for IFDs, for example, high‐dose corticosteroids, prolonged episodes of neutropenia, intensive‐timing treatment for acute myeloid leukaemia, acute and chronic graft‐versus‐host disease (GvHD), and increased age at diagnosis [7].

While mould infections, such as invasive aspergillosis (IA), are associated with a higher one‐year mortality rate compared to yeast infections [3], invasive *Candida* infections are more prevalent [1]. In fact, *Candida* spp. accounted for 50% of invasive fungal infections in a Canadian acute myeloid leukaemia patient population [5]. Chronic disseminated candidiasis (CDC) is a rare yet severe form of invasive candidiasis that occurs after prolonged neutropenic episodes in patients with hematologic malignancies or after HSCT, and its diagnosis and clinical management remain challenging [8, 9, 10, 11, 12]. Reported incidence rates of CDC in patients with acute leukaemia range from 2% to 9% [10, 12, 13]; however, reliable information on incidence rates in the paediatric cancer patient population is lacking. In conditions of chemotherapy‐induced neutropenia and mucosal damage, CDC is believed to be caused by the entry and dissemination of *Candida* spp. from the digestive tract through the portal venous system to end organs [13, 14]. Definite diagnosis of CDC by histology often is challenging, and it therefore often remains a probable IFD per definition [10, 15]. Diagnostic criteria for a probable candidiasis comprise the presence of host factors such as recent history of neutropenia or prolonged steroid treatment, clinical features like small target‐like abscesses (bull's‐eye lesions) in, for example, liver or spleen on imaging, and mycological evidence through positive molecular assays or positivity of Beta‐D‐Glucan (BDG) in patient serum [15]. BDG is a polysaccharide cell wall component of multiple fungal genera including *Aspergillus, Candida, Fusarium*, and *Pneumocystis* that can be detected and quantified in biological specimens by its activation of factor G from the horseshoe crab coagulation cascade [16, 17]. BDG therefore is a useful but nonspecific biomarker in patients with suspected IFDs and *Pneumocystis* pneumonitis.

Little is known about the utility of BDG for diagnosis and response monitoring of CDC. We therefore describe the diagnosis and course of disease of paediatric cancer patients diagnosed with suspected chronic disseminated candidiasis as well as the utility and pitfalls of serum BDG in proven and probable CDC for diagnosis and monitoring of treatment responses.

## 2 Patients and Methods

The study was a retrospective single‐center observational cohort study. The study population included paediatric cancer patients < 20 years of age with a diagnosis of proven or probable CDC [15]—receiving treatment or infectious disease consultation at the Department of Haematology and Oncology, University Children's Hospital Münster between January 2013 and December 2024. Written informed consent for data collection and analysis was obtained within the consent procedure for cancer treatment and specialised medical care approved by the local institutional review boards.

The Department of Paediatric Haematology and Oncology of the University Children's Hospital of Muenster is located in the Northwest of Germany and provides specialised care for the entire spectrum of paediatric haematological and oncological disorders. Each year, 140 to 160 patients with a new diagnosis of cancer, 20 to 40 patients with recurrent cancer, and approximately 35 patients scheduled to undergo allogeneic HCT are admitted, accounting for approximately 1200 hospital admissions and close to 15,000 outpatient or day clinic contacts [18]. Treatment regimens follow recommendations and protocols of national and international registries and study groups [19, 20]. As standard of care, > 95% of patients receive a central venous catheter prior to the initiation of chemotherapy. All patients receive trimethoprim/sulfamethoxazole 8 mg/kg body weight twice weekly as prophylaxis of *Pneumocystis jirovecii* pneumonia and topical polyenes for prevention of oropharyngeal candidiasis. Specialised Infectious Disease supportive care is provided by the Department's Infectious Disease Research Program. Indications for and use of systemic antifungal prophylaxis [6, 21] and evaluation and management of suspected or proven infections follow standard recommendations and guidelines [6, 22, 23, 24, 25]. Systemic antibacterial prophylaxis is not routinely administered.

Cases of CDC were identified, prospectively followed, and documented by the Head of the Department's Infectious Diseases Research Program (AHG). Diagnostic work‐up beyond standard procedures recommended for initial work‐up of a suspected infection, including specialised imaging and additional or repeat microbiological or biochemical diagnostics, was guided by clinical and laboratory findings. Skin biopsies were taken upon the appearance of skin lesions, and biopsies of solid organs were performed in case of acceptable benefit–risk assessment or in case of treatment failure. Serum levels of BDG were obtained serially (> one sample) in all cases of imaging findings suggestive of CDC. BDG in serum was measured by a commercially available assay (Fungitell; Associates of Cape Cod, MA, USA) without fixed schedule at the discretion of the responsible physicians at 5 and 10 time points over a period of 200 to 400 days post diagnosis. Probable and proven CDC were defined according to the revised and updated IFD definitions of the EORTC/MSGERC consensus group 2020 [15].

For the purpose of analysis, clinical, radiographic, and microbiological data of each patient were retrospectively extracted from the medical information system for a minimum of 365 days post CDC diagnosis. Data collection was accomplished by a pseudonymized standardised case report form. Granulocytopenia was defined as an ANC of < 500/μL, and fever was defined as a single oral temperature ≥ 38.3°C or temperatures ≥ 38.0°C during a 1‐h period [26]. The diagnosis of a fungal blood stream infection (fungemia) was based on the detection of the organism in ≥ 1 blood culture bottle, and all IFD definitions followed those set forth by the EORTC/MSGERC consensus group 2020 [15]. Mucositis was defined according to the Common Toxicity Criteria (CTC) for adverse events version 4.03 [27].

Data were tabulated and analysed descriptively. The primary endpoint of the study was to describe the dynamics of BDG in serum in BDG‐positive patients from diagnosis through the end of treatment and to assess the utility of BDG serum levels for monitoring of response to antifungal treatment. Secondary endpoints included the diagnostic validity of BDG in CDC; the correlation with clinical, radiologic, and microbiological variables; antifungal treatment and treatment duration; treatment response by resolution of imaging findings; and survival through the end of treatment.

## 3 Results

During the study period, a total of five patients (four girls, one boy; 4–18 years old) with an initial diagnosis of proven or probable CDC were identified and prospectively followed (Table 1). Four patients had acute lymphoblastic leukaemia (ALL) in complete cytomorphological remission, and one patient was diagnosed with Ewing Sarcoma and had stable disease at the time of probable CDC diagnosis. All patients received dose‐intense antineoplastic chemotherapy on treatment protocols open at the time of diagnosis, and all four ALL patients received additional high‐dose glucocorticosteroids as part of their treatment. All patients had a permanent central venous catheter in place, and all experienced periods of severe granulocytopenia and developed mucositis within the last 4 weeks prior to the diagnosis of CDC. All patients with CDC presented with fever before and after the diagnosis of CDC. Other recorded clinical symptoms included hepatomegaly (all patients), splenomegaly (four patients), nausea, vomiting, and abdominal pain (all patients), and skin lesions (one patient). C‐reactive protein (CRP) levels in serum were elevated in all patients, with median values ranging from 4.9 to 20.6 mg/dL during the course of CDC in all patients. Elevated alkaline phosphatase levels were present only in one patient (median 362 U/L) (Table 1).

**TABLE 1:** Demographics, risk factors, and clinical presentation in five patients with an initial diagnosis of probable or proven chronic disseminated candidiasis (CDC).

Characteristics	Patient 1	Patient 2	Patient 3	Patient 4	Patient 5
Age at diagnosis (years)	9	11	4	5	18
Sex	Female	Male	Female	Female	Female
Malignancy	B‐ALL	T‐ALL	B‐ALL	B‐ALL	Ewing Sarcoma
Chemotherapy regimen	AIEOP‐BFM‐ALL 2009, Protocol Ib	AIEOP‐BFM‐ALL 2009, Protocol IIa	AIEOP‐BFM ALL 2017, Protocol Ib	AIEOP‐BFM ALL 2017, cons. B	Ewing 2008, 6th VIDE—cycle
CVC	+	+	+	+	+
Corticosteroids	+	+	+	+	−
Granulocytopenia	+	+	+	+	+
Mucositis	+	+	+	+	+
Status of underlying disease	Complete remission	Complete remission	Complete remission	Complete remission	Stable disease
CDC clinical findings					
Fever	+	+	+	+	+
Hepatomegaly	+	+	+	+	+
Splenomegaly	−	+	+	+	+
Nausea, vomiting	+	+	+	+	+
Abdominal pain	+	+	+	+	+
Skin lesions	−	+	−	−	−
Laboratory findings (median, range)					
CRP (mg/dL)	20.6 (0.5–30.3)	11 (0.5–28.5)	12.1 (0.5–34.1)	4.9 (< 0.3–24.4)	13.1 (0.5–28.1)
Alk Phos (U/L)	226 (87–855)	109 (72–234)	362 (40–1168)	182 (135–238)	138 (99–270)

Abbreviations: AlkPhos, alkaline phosphatase; ALL, acute lymphoblastic leukaemia; CRP, C‐reactive protein; CVC, central venous catheter.

In all patients, spleen lesions were present by sonography and magnetic resonance imaging (MRI). Additional lesions documented by sonography and MRI were found in the liver (four patients), the lung (four patients), the central nervous system (two patients), and the kidney (one patient). Infectious myocarditis was suspected by laboratory and functional studies (echocardiography) in one patient (patient 3). Microbiologic results revealed a positive blood culture ( *C. albicans* ) in only one of the five patients who also had a positive skin culture for the same organism from one of his skin lesions. Oropharyngeal swab cultures were documented in three patients, and *C. albicans* could be detected in two of them. No cultures from stool or perianal swabs were documented, and no screening for *P. jirovecii* pneumonia (PJP) due to the absence of respiratory signs and symptoms (Table 2). Biopsies of lesions other than those of the skin were performed in two patients: while a kidney biopsy of one patient (patient 3) remained without histological and cultural evidence for a fungal or bacterial pathogen, a liver biopsy in the patient with Ewing Sarcoma (patient 5) performed 5 months after diagnosis of probable CDC because of lack of clinical and radiographic improvement revealed disseminated aspergillosis by *A. fumigatus*; repeated galactomannan testing in serum had been negative at the time of initial presentation. However, there was a preceding lung lesion that in retrospect may have been the starting point of disseminated aspergillosis in this patient (Table 2).

**TABLE 2:** Imaging findings and microbiological results in five patients with an initial diagnosis of probable or proven chronic disseminated candidiasis (CDC).

Characteristics	Patient 1	Patient 2	Patient 3	Patient 4	Patient 5
Lesions on radiologic investigations					
Liver	+	−	+	+	+
Spleen	+	+	+	+	+
Kidney	−	−	+	−	−
Lung	+	+	+	+	−
CNS	−	+	−	−	+
Mycological data at diagnosis					
Microbiologic results					
Blood culture	Negative	*C. albicans*	Negative	Negative	Negative
Oral swab culture	Negative	*C. albicans*	*C. albicans*	Not done	Not done
Skin lesion culture	Not done	*C. albicans*	Not done	Not done	Not done
Biopsy results					
Tissue	Not done	Skin (pos)	Kidney (neg)	Not done	Liver (pos)
BDG levels pg/mL					
At diagnosis	> 500	112	> 500	> 500	212.2
At resolution of lesions	11	71	< 60	< 60	No resolution
Range	11 to > 500	71 to > 500	< 60 to > 500	< 60 to > 500	212.2 to 523.4
Number of samples	8	5	10	7	5

Abbreviations: BDG, beta‐D‐glucan; CNS, central nervous system.

**FIGURE 1:**
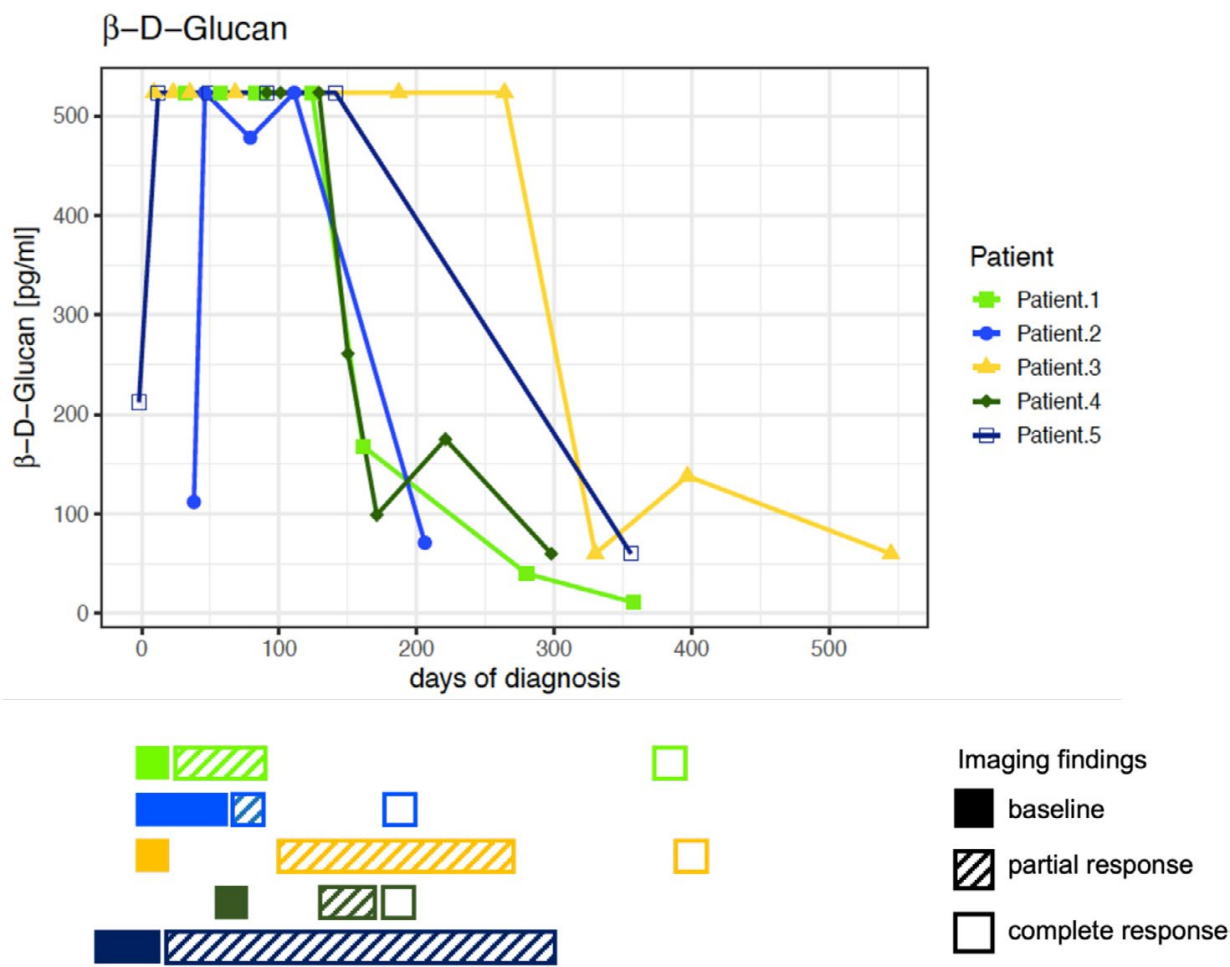
Course of β‐D‐Glucan (BDG) in serum over time in five patients with an initial diagnosis of probable or proven chronic disseminated candidiasis (CDC) and corresponding imaging findings. Note that patient 5 was diagnosed at 5 months post start of treatment with disseminated invasive aspergillosis by liver biopsy. BDG concentrations were quantified from 20 to a maximum of 500 ng/mL. Values exceeding 500 ng/mL were reported as ≥ 500 ng/mL. A value of ≥ 60 ng/mL was considered a positive test result.

In all patients, BDG in serum was continuously determined from the moment of suspected CDC (35 time points in total for all patients). Highly elevated BDG concentrations of > 500 pg/mL were observed in all five patients during the course of the disease; however, at the diagnosis of CDC, such highly elevated BDG concentrations were apparent in only three patients. BDG concentrations in serum remained elevated for a mean duration of 427 days (range: 208 to 787 days), were independent of clinical signs and symptoms, and decreased upon resolution of imaging findings in the four leukaemia patients (Figure 1; Table 2).

Treatment consisted of intravenous liposomal amphotericin B and/or caspofungin, followed by oral step‐down to fluconazole for a total duration of between 184 to > 365 days, respectively. One patient each received additional voriconazole or posaconazole, respectively, and patient five was treated with posaconazole after the diagnosis of invasive aspergillosis. All four patients with proven or probable CDC (patients one to four) are alive after a median follow‐up of 3042 days. Patient five died with disseminated aspergillosis due to refractory Ewing sarcoma 787 days after the presumptive diagnosis of CDC (Table 3).

**TABLE 3:** Antifungal treatment and outcome in five patients with an initial diagnosis of probable or proven chronic disseminated candidiasis (CDC).

Characteristics	Patient 1	Patient 2	Patient 3	Patient 4	Patient 5
Antifungal therapy					
Liposomal AMB	+	+	+	+	+
Caspofungin	+	+	+	−	+
Fluconazole	+	+	+	+	+
Voriconazole	+	−	−	−	−
Posaconazole	−	−	−	+	+
Therapy duration (days)	381	184	585	303	787
Response					
Time to resolution of imaging findings (days)	380	208	395	165	No resolution
Time to normalisation of BDG levels (days)	280	208	545	315	No normalisation
Outcome					
Alive/deceased	Alive	Alive	Alive	Alive	Deceased^b^
Follow up^a^ (days)	4377	4290	2165	1337	787

Abbreviation: AMB, amphotericin B.

^a^ Starting with first day of antifungal treatment.

^b^ Due to recurrent underlying disease.

## 4 Discussion

The observations made in this small case series suggest that BDG in serum can be a useful adjunct to diagnosis and response monitoring of CDC in paediatric cancer patients. Serial BDG serum levels showed a correlation with the dynamics of imaging findings and the response to antifungal treatment; they ultimately normalised with the resolution of pathologic imaging findings in the four leukaemia patients. However, as exemplified by the patient with Ewing sarcoma and a final diagnosis of invasive aspergillosis, BDG is a non‐specific fungal biomarker whose diagnostic validity needs to be scrutinised in patients who do not respond to treatment as expected.

While BDG has become a valuable biomarker in the diagnosis of PJP in immunocompromised patients [28, 29, 30], its usefulness for diagnosing other IFDs is met with limited evidence due to moderate accuracy [30]. This is particularly true in the paediatric setting, where studies evaluating the efficacy of BDG for diagnosing IFDs in at‐risk patients are scarce. Few case series have been published that included paediatric patients with underlying diseases other than cancer, hematologic malignancies, or HCT [31, 32, 33, 34, 35, 36]. More recent comprehensive reviews and systematic meta‐analyses [37, 38] identified a limited number of studies investigating the diagnostic value of BDG for the diagnosis of IFDs in paediatric cancer patients [39, 40, 41, 42, 43]. Although sensitivity rates of the BDG assay of up to 82% were observed [42], overall poor positive predictive values and lack of specificity curtail its application for IFD diagnosis [39, 41, 42, 43]. *Guitard* et al. could demonstrate improved sensitivity and specificity of BDG testing in patients who did not receive antifungal treatment prior to testing [40]; testing before antifungal treatment, however, might be challenging in high‐risk paediatric leukaemia and HCT patients as most of them receive antifungal prophylaxis as recommended by international guidelines [17]. In addition, the appropriate cutoff value for a positive BDG test in children is yet unclear: *Smith* and colleagues reported in 2007 higher baseline BDG levels in children not at risk for IFDs than those previously found in adult patients [33]. Nevertheless, higher baseline levels in that study might have resulted from falsely elevated BDG concentrations that were due to confounders such as patients receiving coagulation factors or albumin. In a prospective cohort study of paediatric patients undergoing HCT, the BDG cutoff value was observed to be age‐independent. The analysis indicated a cutoff value of the Fungitell assay of 70 pg/mL which is higher than the cutoff value suggested by a meta‐analysis in adult patients but lower than the one recommended by the manufacturer [42]. Overall, the optimal BDG cutoff value in children seems to be dependent on the indication and patient group, as demonstrated by a retrospective study in neonates where the optimal cutoff value to distinguish invasive candidiasis was 125 pg/mL [44]. Despite these challenges, the revised EORTC consensus definitions propose a Fungitell BDG assay cutoff value of 80 pg/mL for both adult and paediatric patients [15]. In contrast, based on the lack of statistical power of BDG in the diagnosis of IFDs in paediatric cancer and HCT patients and the limited data in patients with prolonged neutropenia [38, 40, 41, 42], there was a consensus against recommending BDG use for early detection of IFDs in this population in the latest ECIL guidelines [6].

Due to its occurrence in several diverse fungal species, BDG lacks specificity for defined IFDs. This is illustrated by the analysis of *Koltze* and colleagues, which showed elevated BDG serum concentrations in patients with fusariosis and invasive aspergillosis [42], but also by our patient with Ewing sarcoma and a final diagnosis of invasive aspergillosis, in whom imaging findings and highly elevated BDG levels initially suggested CDC. However, very limited data exist thus far on the utility of BDG in the diagnosis of CDC in paediatric cancer patients, which is likely due to the scarcity of cases in the era of antifungal prophylaxis in high‐risk patients. In addition, most investigations in paediatric cancer patients have focused on the value of BDG for early detection of IFDs, rather than on its utility in monitoring responses to antifungal treatment. *Guitard* and colleagues retrospectively investigated the utility of BDG in the diagnosis and follow‐up of invasive candidiasis in adult and paediatric patients with hematologic malignancies and found an overall poor sensitivity of the test but a correlation between the level of BDG at diagnosis and the outcome of candidemia. In that study, BDG negative results were obtained 2 to 6 months before recovery of the CT‐scan lesions in all CDC cases [45]. This is largely in line with our observations on the time course of BDG serum levels and their decline with response to treatment as assessed by imaging. Our data suggest that serial measurement of BDG serum concentrations may be integrated into therapeutic decision making and used as a marker for response to antifungal treatment in CDC patients, acknowledging its limited diagnostic validity and the need for re‐assessment in the case of absent response or deterioration. Due to the markedly limited number of patients and the retrospective character of our observations, the generalizability of these conclusions is restricted, and further studies will be needed to determine the diagnostic value and utility of BDG in paediatric CDC patients.

Last but not least, it is important to acknowledge the existence of multiple confounding factors that may affect BDG serum levels, as comprehensively reviewed in 2020 by Finkelman [46]. Consideration should be given to potential iatrogenic sources of contamination, including certain intravenously administered drug formulations, blood‐derived products, surgical materials, enteral feeding solutions, systemic bacterial infections, and severe mucositis [46, 47]. For instance, some medications may contain excipients with BDG contaminants [48]; blood fractionation products such as intravenous immunoglobulins (IVIG) or serum albumin may be exposed to BDG during filtration through cellulose membranes [49, 50, 51]; and surgical materials such as sponges or gauze can release high levels of BDG when applied invasively or during operative procedures [52, 53, 54]. BDG titers have been observed to decline rapidly following IVIG administration or surgery, supporting the interpretation that such elevations may reflect transient, iatrogenic contamination rather than invasive fungal disease [46, 49, 55]. While parenterally administered antibiotics were previously suspected as a potential source of BDG elevation [47, 56], their clinical relevance in this context is now considered negligible due to the high dilution factor associated with their low injection volumes [57, 58]. Another critical confounder is the intestinal translocation of BDG resulting from impaired mucosal barrier integrity. This mechanism is particularly relevant in the setting of chemotherapy‐induced mucositis or in patients with enterococcal bloodstream infections or intestinal colonisation by *Enterococcus* species [40, 46, 59]. Supporting this, patients receiving chemotherapy who developed mucositis—yet had no evidence of IFD—demonstrated significantly elevated BDG levels compared to those without mucosal damage and without IFD [60]. Addressing these potential confounders is therefore imperative when utilising BDG as a diagnostic tool for IFDs, underscoring the importance of careful consideration in its clinical application, particularly in cases where treatment responses deviate from expectations.

In conclusion, detection of BDG serum levels may be useful for diagnosis and monitoring responses to antifungal treatment in paediatric CDC patients. However, its non‐specific nature and susceptibility to confounding factors warrant cautious interpretation, particularly in patients who do not respond to treatment as expected.

## Author Contributions

**Katharina F. Körholz:** writing – review and editing, conceptualization, data curation, formal analysis, visualization, writing – original draft, investigation, methodology. **Marc T. Hennies:** methodology, writing – review and editing, data curation. **Heidrun Herbrüggen:** methodology, investigation, writing – review and editing. **Katja Krämer:** data curation, methodology, writing – review and editing, investigation. **Martina Ahlmann:** methodology, investigation, writing – review and editing. **Birgit Fröhlich:** investigation, methodology, writing – review and editing. **Frieder Schaumburg:** methodology, investigation, writing – review and editing. **Thomas Wiesel:** methodology, investigation, writing – review and editing. **Peter M. Rath:** methodology, investigation, writing – review and editing. **Andreas H. Groll:** conceptualization, formal analysis, visualization, writing – original draft, writing – review and editing, supervision, investigation, methodology.

## Disclosure

The authors have nothing to report.

## Ethics Statement

Written informed consent for off label drug use, data collection and analysis was obtained within the consent procedure for cancer treatment, HCT and specialised medical care approved by the local institutional review board. Informed consent to be included in this analysis was waived as the study did not involve additional procedures to the standardised clinical protocols and all data were treated in an anonymous fashion.

## Conflicts of Interest

A.H.G. has received grants from Gilead, Merck, Sharp & Dohme and Pfizer and has served as consultant to Amplyx, Astellas, Basilea, F2G, Gilead. Merck, Sharp & Dohme, Pfizer, Scynexis, and Mundipharma. The other authors declare no conflicts of interest.

## Data Availability

The data that support the findings of this study are available from the corresponding author upon reasonable request.
